# Decreased peripheral CD4^+^/CD8^+ ^lymphocytes and poor prognosis in aged sepsis

**DOI:** 10.1186/cc10612

**Published:** 2012-03-20

**Authors:** S Inoue, K Utsunomiya-Suzuki, S Morita, T Yamagiwa, S Inokuchi

**Affiliations:** 1 Tokai University, Kanagawa, Japan

## Introduction

Aging is a significant factor and is associated with a poor prognosis in sepsis; however, the mechanism of immunological changes in aged sepsis is still unclear. The purpose of this study was to clarify the immunological changes in sepsis of aged patients.

## Methods

Forty-four septic patients and 48 gender-matched healthy volunteers were prospectively enrolled in the study, which included the following investigations: (1) The SOFA score and clinical outcome were compared between adult sepsis (<65 years of age) and older adult sepsis (≥65 years of age). (2) Blood samples were collected from septic and control volunteers. Separated peripheral blood mononuclear cells were stained with CD4, CD8, programmed death-1 (PD-1), CD28, and CD62L antibodies and analyzed by flow cytometry, and serum was used to measure cytokine concentrations by using multiplex bead assay. Values were compared among four groups: normal adult (<65 years of age), normal older adult (≥65 years of age), adult sepsis (<65 years of age), and older adult sepsis (≥65 years of age) groups.

## Results

(1) No differences in SOFA scores were observed between adult sepsis (*n *= 19, 39 years) and older adult sepsis (*n *= 25, 78 years), but 3-month survival in older adult sepsis was significantly decreased compared with that in adult sepsis (36% vs. 4%, *P *< 0.05). (2) Population of CD8^+ ^T cells in normal older adults was significantly less than that in normal adults (1.5 × 10^5 ^vs. 5.7 × 10^4^/ml, *P *< 0.01), and percentage of PD-1^+^CD8^+ ^T cells in the older adult sepsis group was significantly greater than that in the normal older adult group (40% vs. 29%, *P *< 0.01). Population of CD4^+^, CD62L^+^CD4^+^, and CD28^+^CD4^+ ^T cells in the older adult sepsis group was significantly less than that in the normal older adult group (*n *= 26, 80 years) (1.8 × 10^5 ^vs. 5.9 × 10^4^/ml, 1.6 × 10^5 ^vs. 5.4 × 10^4^/ml, and 1.6 × 10^5 ^vs. 4.4 × 10^4^/ml, respectively; *P *< 0.01); however, these values did not differ between the adult sepsis and normal adult (*n *= 22, 39 years) groups. Serum IL-12 level in older adult sepsis was increased when compared with that in the other three groups (*P *< 0.01).

## Conclusion

Poor prognosis in older adult sepsis may be related to both preexisting decrease of CD8^+ ^T cells with aging and loss of CD4^+ ^T cells with sepsis.

